# Real-world characteristics, modern antidiabetic treatment patterns, and comorbidities of patients with type 2 diabetes in central and Eastern Europe: retrospective cross-sectional and longitudinal evaluations in the CORDIALLY^®^ study

**DOI:** 10.1186/s12933-022-01631-4

**Published:** 2022-10-08

**Authors:** Martin Prázný, Lyudmila Suplotova, Janusz Gumprecht, Zdravko Kamenov, Tibor Fülöp, Alexey Medvedchikov, Doron Rosenzweig, Milos Aleksandric

**Affiliations:** 1 3rd Department of Internal Medicine, 1st Faculty of Medicine, Charles University, Prague, Czech Republic; 2 Tyumen State Medical University, Tyumen, Russian Federation; 3 Department of Internal Medicine, Diabetology and Nephrology in Zabrze, Faculty of Medical Sciences in Zabrze, Medical University of Silesia, Katowice, Poland; 4 Department of Internal Medicine, Medical University Sofia, Sofia, Bulgaria; 5 Clinic of Endocrinology, University Hospital Alexandrovska, Sofia, Bulgaria; 6 Department of Cardiology and Heart Surgery, Medical and Health Science Center, University of Debrecen, Debrecen, Hungary; 7 Boehringer Ingelheim RCV GmbH and Co. KG, Vienna, Austria

**Keywords:** Cardiovascular disease (CVD), Cardiovascular outcomes trials (CVOTs), Cardiovascular safety, Chronic kidney disease (CKD), Dipeptidyl peptidase-4 inhibitors (DPP4i), Glucagon-like peptide-1 receptor agonists (GLP-1 RA), Glucose-lowering drug, Type 2 diabetes, Sodium-glucose cotransporter-2 inhibitors (SGLT2i)

## Abstract

**Background:**

Guidelines from 2016 onwards recommend early use of SGLT2i or GLP-1 RA for patients with type 2 diabetes (T2D) and cardiovascular disease (CVD), to reduce CV events and mortality. Many eligible patients are not treated accordingly, although data are lacking for Central and Eastern Europe (CEE).

**Methods:**

The CORDIALLY non-interventional study evaluated the real-world characteristics, modern antidiabetic treatment patterns, and the prevalence of CVD and chronic kidney disease (CKD) in adults with T2D at nonhospital-based practices in CEE. Data were retrospectively collated by medical chart review for patients initiating empagliflozin, another SGLT2i, DPP4i, or GLP-1 RA in autumn 2018. All data were analysed cross-sectionally, except for discontinuations assessed 1 year ± 2 months after initiation.

**Results:**

Patients (N = 4055) were enrolled by diabetologists (56.7%), endocrinologists (40.7%), or cardiologists (2.5%). Empagliflozin (48.5%) was the most prescribed medication among SGLT2i, DPP4i, and GLP-1 RA; > 3 times more patients were prescribed empagliflozin than other SGLT2i (10 times more by cardiologists). Overall, 36.6% of patients had diagnosed CVD. Despite guidelines recommending SGLT2i or GLP-1 RA, 26.8% of patients with CVD received DPP4i. Patients initiating DPP4i were older (mean 66.4 years) than with SGLT2i (62.4 years) or GLP-1 RA (58.3 years). CKD prevalence differed by physician assessment (14.5%) or based on eGFR and UACR (27.9%). Many patients with CKD (≥ 41%) received DPP4i, despite guidelines recommending SGLT2is owing to their renal benefits. 1 year ± 2-months after initiation, 10.0% (7.9–12.3%) of patients had discontinued study medication: 23.7–45.0% due to ‘financial burden of co-payment’, 0–1.9% due to adverse events (no patients discontinued DPP4i due to adverse events). Treatment guidelines were ‘highly relevant’ for a greater proportion of cardiologists (79.4%) and endocrinologists (72.9%) than diabetologists (56.9%), and ≤ 20% of physicians consulted other physicians when choosing and discontinuing treatments.

**Conclusions:**

In CORDIALLY, significant proportions of patients with T2D and CVD/CKD who initiated modern antidiabetic medication in CEE in autumn 2018 were not treated with cardioprotective T2D medications. Use of DPP4i instead of SGLT2i or GLP-1 RA may be related to lack of affordable access, the perceived safety of these medications, lack of adherence to the latest treatment guidelines, and lack of collaboration between physicians. Thus, many patients with T2D and comorbidities may develop preventable complications or die prematurely.

*Trial registration* NCT03807440.

**Supplementary Information:**

The online version contains supplementary material available at 10.1186/s12933-022-01631-4.

## Background

Diabetes affects about 537 million people worldwide and is expected to increase to 784 million by 2045 [1]. Cardiovascular disease (CVD), the most common cause of mortality for people with type 2 diabetes (T2D), may account for more than 50% of deaths in this patient population [2]. The life expectancy of a 60 year-old man with T2D and either a history of stroke or myocardial infarction may be shortened by about 12 years [3]. In addition, risk of myocardial infarction and all-cause mortality are substantially higher in patients with diabetes and comorbid chronic kidney disease (CKD), compared with diabetes or CKD alone or the absence of both diseases [4].

Since 2015, several CardioVascular Outcomes Trials (CVOTs) have demonstrated that sodium-glucose cotransporter-2 inhibitors (SGLT2i) and glucagon-like peptide-1 receptor agonists (GLP-1 RA) provided significant cardiorenal benefits, compared with placebo, for patients with T2D [5–11]. CVOTs of dipeptidyl peptidase-4 inhibitors (DPP4i) confirmed cardiovascular (CV) safety, but without significant CV benefits [12–14] (and showed a potentially increased risk of hospitalisation for heart failure with saxagliptin) [13]. Since 2019, significant cardiorenal benefits have been demonstrated with SGLT2i in patients with heart failure (with reduced or preserved ejection fraction) or CKD, either with comorbid T2D or regardless of T2D status [15–19]. Cardiorenal benefits of SGLT2i, compared with DPP4i and other glucose-lowering drugs, have also been demonstrated in large real-world studies [20–23].

Based on these studies, there has been a paradigm shift in national and international T2D treatment guidelines; SGLT2i or GLP-1 RA are recommended either as first-line therapy or after metformin for patients with atherosclerotic CVD or at high CV risk, while SGLT2i is preferred after metformin for patients with heart failure or CKD [24, 25]. Early use of these agents in the treatment of T2D, particularly in patients with comorbid CVD to reduce major adverse CV events and CV mortality, has been recommended in guidelines from 2016 onwards [26, 27]. However, despite these clear recommendations, recent real-world studies in Europe, North America, Latin America, Asia, and Australia demonstrate that a significant number of patients who meet the criteria for early treatment with cardioprotective T2D medications are not treated accordingly [28–32]. For instance, in the global CAPTURE study, only 21.5% of patients with comorbid T2D and CVD received prescriptions for SGLT2i or GLP-1 RA in 2019 [32]. Regarding patients with T2D and impaired renal function, a real-world study in the UK demonstrates that 3.2% and 8.8% of patients with estimated glomerular filtration rate (eGFR) of 45 to < 60 ml/min and 60 to < 90 ml/min, respectively, were prescribed SGLT2i between January 2018 and March 2019 [33].

Knowing the characteristics of patients initiating different T2D treatments should lead to a better understanding of the prescription patterns of modern antidiabetic medications. However, in Central and Eastern European (CEE) countries, there is a lack of available information on these prescription patterns and patient profiles, including T2D characteristics and demographics. In the CAPTURE study, the only CEE country was the Czech Republic [32], while other European countries in relevant studies were in the west of the continent [28, 31, 32]. Data are also lacking in CEE countries on the prevalence of comorbidities (such as CVD and CKD) affecting the use of modern antidiabetic medications, socioeconomic factors that could limit treatment initiation, and the medical and socioeconomic factors related to treatment discontinuation. Here, we report cross-sectional and longitudinal evaluations from CORDIALLY, a large, real-world, non-interventional study that was conducted with the intention of filling this information gap, based on data from patients with T2D newly initiating empagliflozin, another SGLT2i, DPP4i, or GLP-1 RA in autumn 2018.

## Methods

### Study design and patients

CORDIALLY was a retrospective cross-sectional and longitudinal non-interventional study evaluating the characteristics, modern antidiabetic treatment patterns, and cardiorenal comorbidities of patients with T2D, under routine clinical conditions, in five CEE countries (Bulgaria, Czech Republic, Hungary, Poland, and Russia) (ClinicalTrials.gov: NCT03807440). An overview of CORDIALLY is shown in Additional file 1: Figure S1.

Eligible patients were adults (≥ 18 years of age) with a diagnosis of T2D, who newly initiated (first ever use) empagliflozin, another SGLT2i, DPP4i, or GLP-1 RA at baseline (September–December 2018), and provided written informed consent.

All data were collected retrospectively, after gaining written informed consent, by medical chart review. Healthcare professionals (HCPs) checked that patients met all eligibility criteria and subsequently transferred their data from baseline and from 1 year ± 2 months after baseline into an electronic case report form (eCRF).

Several procedures were adopted to reduce potential bias and confounders. Data were collected for all eligible patients in diabetologist, endocrinologist, and cardiologist office-based (nonhospital) practices. The treatment decision was taken before and independently from deciding to enrol a patient in this non-interventional study and was at the discretion of the HCP. Study sites were randomly selected by the study sponsor from a list of proposed sites, which included 50% more sites than were finally included in the study. Study sites were eligible if HCPs had access to, and could prescribe, at least 2 of the T2D drug classes of interest.

The study complied with the principles of the Declaration of Helsinki.

### Objectives and outcomes

The primary objective was to describe and compare the baseline characteristics of adults with T2D, when initiating empagliflozin, another SGLT2i, DPP4i or GLP-1 RA on top of current antidiabetic treatment, by different HCP specialties in CEE countries. Primary outcomes included demographics and clinical parameters relevant to T2D, CVD, and CKD. A comprehensive list of outcomes is provided in Additional file 1: Table S1.

Secondary objectives, using data collected at baseline, were to describe the prevalence of CVD, CKD, and associated risk factors; to compare treatment use in patients with and without CVD; and to describe associations between socioeconomic parameters and treatment decisions. CVD was defined as documented acute myocardial infarction, cardiology intervention (percutaneous coronary intervention [PCI], coronary artery bypass graft [CABG]), ischemic heart disease, congestive heart failure, peripheral arterial disease, or stroke. Another secondary objective, using data from 1 year ± 2 months after baseline, was to assess the discontinuation rate, primary reasons for discontinuation, and duration of treatment with SGLT2i, DPP4i, and GLP-1 RA.

HCPs provided reasons for choosing the study medication (rated as ‘not relevant at all’, ‘moderately relevant’ or ‘highly relevant’) from the following options: glycated haemoglobin (HbA1c) lowering, weight loss, CV risk reduction, favourable adverse event (AE) profile, simple dosing/administration, guideline recommendations. HCPs stated whether or not other HCPs were involved in treatment decisions regarding prescription and discontinuation of T2D study medications and, if so, the medical specialty was to be specified. No AEs had to be recorded in the eCRF, except in relation to discontinuation of T2D study medication.

### Sample size

No formal sample size calculation was conducted. A sample size of approximately 4000 patients was derived by the feasibility of recruiting an adequate number of patients to address the objectives, considering subgroup analyses, including by HCP specialty.

### Statistical analysis

Baseline data were analysed using the Prescribed Patient Set, comprising all patients with a first prescription of SGLT2i, DPP4i, or GLP-1 RA at baseline. Baseline characteristics were analysed descriptively. In addition, the baseline characteristics were compared by T2D study medication and HCP specialty using χ^2^-test or Fisher’s exact test, if χ^2^-test was not valid, for categorical variables and Kruskal–Wallis test for continuous variables.

Treatment discontinuation rates and reasons were analysed using the Full Analysis Set, which comprised patients from the Prescribed Patient Set with documentation at 1 year ± 2 months post-baseline. To assess the duration of T2D therapy, the time to discontinuation was analysed by Kaplan–Meier estimates and subgroups were compared using log-rank test.

All statistical analyses were performed using SAS 9.4 (SAS Institute Inc., Cary, NC, USA). The analyses were purely explorative, hence no correction for multiple testing was needed. No imputation method was used to substitute missing values.

## Results

### Patient disposition

Of 4083 patients screened, 4055 fulfilled all eligibility criteria, including receiving a first ever prescription of SGLT2i, DPP4i, or GLP-1 RA between September and December 2018, and thus were included in the Prescribed Patient Set for analysis of baseline characteristics (Additional file 1: Figure S2). Most patients (89.2%) had documentation at 1 year ± 2-months post-baseline, and thus were included in the Full Analysis Set for analysis of treatment discontinuation.

**Fig. 1:**
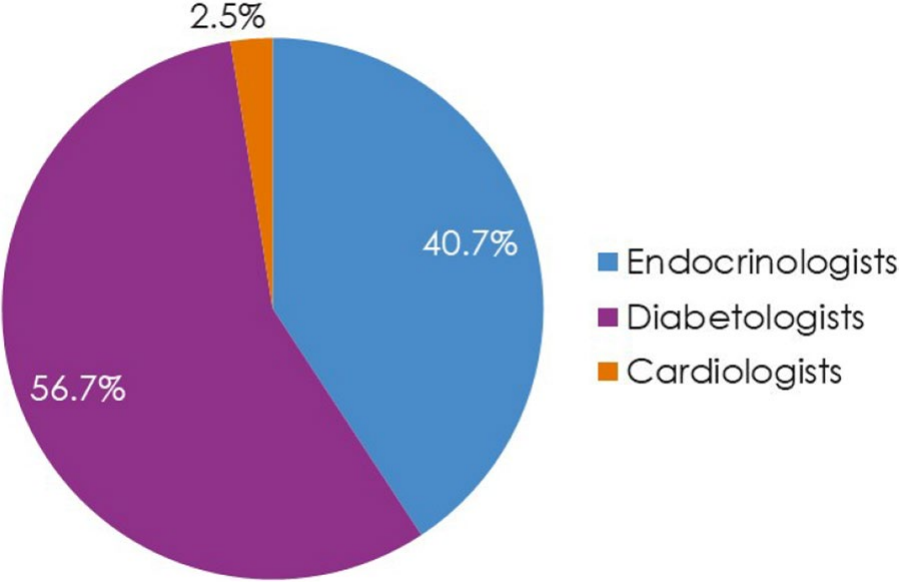
Enrolment by HCP specialty (Prescribed Patient Set). HCP, healthcare professional

Most patients were enrolled by diabetologists (56.7%; N = 2301) and endocrinologists (40.7%; N = 1652), with the remainder (2.5%; N = 102) enrolled by cardiologists (Fig. 1).

Patients were enrolled in Russia (33.5% of all eligible patients), Czech Republic (30.1%), Poland (21.6%), Bulgaria (8.5%), and Hungary (6.3%) (Additional file 1: Figure S2).

Differences in prescription patterns were observed across the five countries (Additional file 1: Figure. S3a). The proportion of patients with an empagliflozin prescription ranged from 33.3% in the Czech Republic (N = 1221) to 70.0% in Poland (N = 876). Prescription ranges for the other study medications were: DPP4i 12.6% (Poland) to 35.4% (Czech Republic), another SGLT2i 12.1% (Hungary) to 21.7% (Bulgaria), and GLP-1 RA 3.0% (Poland) to 16.6% (Czech Republic).

Enrolment by the three HCP specialties also differed by country (Additional file 1: Figure S3b). Patients enrolled by endocrinologists were almost exclusively from Russia (78.8%) and Bulgaria (20.9%), those enrolled by diabetologists were mainly from the Czech Republic (53.0%), Poland (36.1%), and Hungary (10.0%), and those enrolled by cardiologists were mainly from Poland (38.2%), Russia (34.3%), and Hungary (26.5%). The predominance of diabetologists vs endocrinologists among the countries reflects differences in established local practice for specialist care of diabetes; diabetology is a separate specialty from endocrinology in some Eastern European countries [34].

### Primary outcomes: baseline demographics and clinical parameters relevant to T2D, CVD, and CKD

**Fig. 2:**
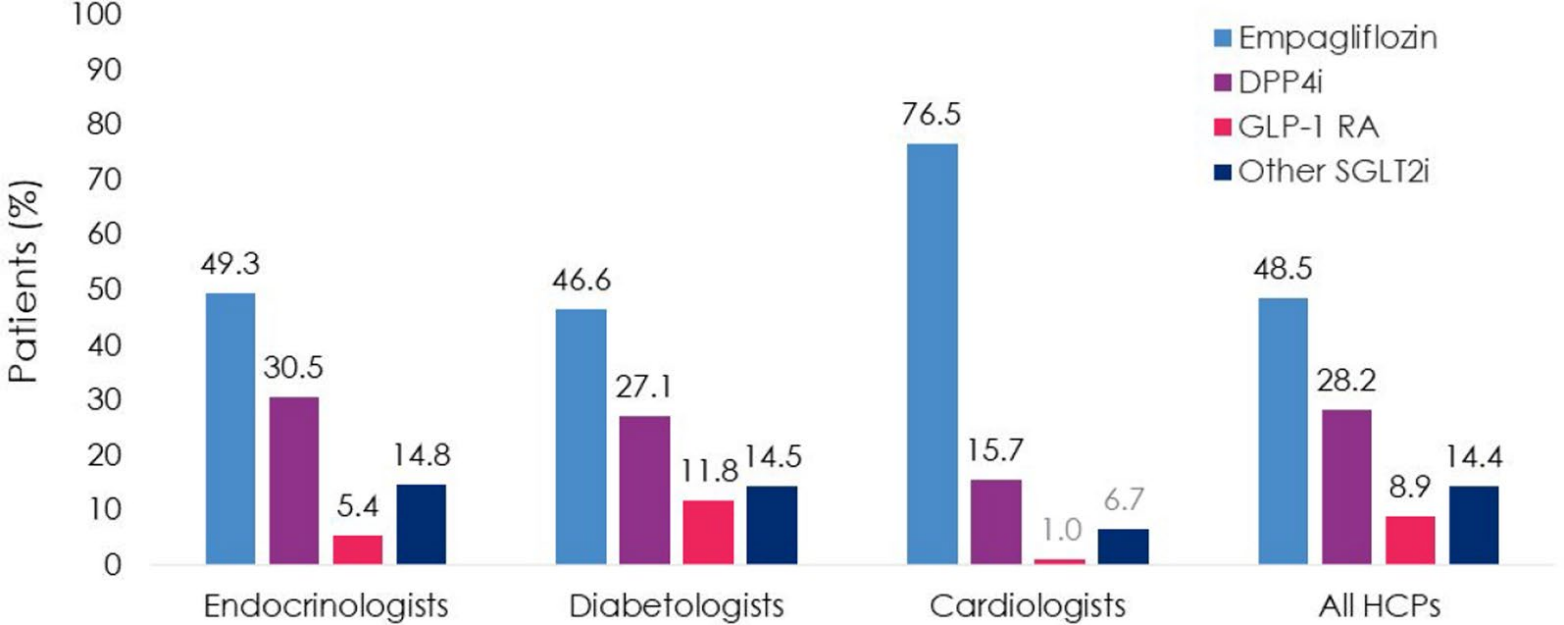
T2D study medication prescriptions (Prescribed Patient Set). DPP4i, dipeptidyl peptidase-4 inhibitor; GLP-1 RA, glucagon-like peptide-1 receptor agonist; HCP, healthcare professional; SGLT2i, sodium-glucose cotransporter-2 inhibitor; T2D, type 2 diabetes

Empagliflozin (48.5%) was the most prescribed study medication in the overall population, followed by DPP4i (28.2%), other SLGT2i (14.4%), and GLP-1 RA (8.9%) (Fig. 2). Cardiologists had the highest percentage of patients with prescriptions of empagliflozin (76.5%), compared with endocrinologists (49.3%) and diabetologists (46.6%) (Fig. 2). Patients were over three times more likely to be prescribed empagliflozin than other SGLT2i when treated by a diabetologist or endocrinologist, and 10 times more likely when treated by a cardiologist (Fig. 2). Most patients with HbA1c ≥ 8.5% were prescribed empagliflozin (51.6%), including 56.9%, 45.6%, and 70.0% of patients treated by an endocrinologist, diabetologist, and cardiologist, respectively.

Patients receiving DPP4i were the oldest (mean 66.4 years; standard deviation [SD] 10.8) and had the lowest body mass index (BMI) (mean 31.1 kg/m^2^; SD 5.3) and HbA1c (mean 8.0%; SD 1.3), while patients receiving GLP-1 RA were the youngest (mean 58.3 years; SD 10.8) and had the highest BMI (mean 36.3 kg/m^2^; SD 6.5) (p < 0.0001) (Table 1a). This was also the case when looking only at each of the endocrinologist or diabetologist subgroups (Table 2a). Mean time since T2D diagnosis, 9.9 years (SD 6.9), was comparable per treatment (p > 0.05) in the overall population (Table 1a) and also in the endocrinologist and diabetologist subgroups (Table 2a). For the cardiologist subgroup (N = 102), cohort sizes per treatment were too small to draw meaningful conclusions about patient characteristics.

**Table 1:** Patient characteristics at baseline in the overall population (Prescribed Patient Set)

Study medication and characteristic	Empa N = 1966	DPP4i N = 1144	GLP-1 RA N = 361	Other SGLT2i N = 584	Total N = 4055
(A) Demographics and clinical parameters relevant to T2D					
Age (years), mean (SD)	62.4 (9.5)	66.4 (10.8)	58.3 (10.8)	62.2 (9.2)	63.1 (10.2)
Female (%)	45.8	55.9	47.6	48.3	49.1
BMI (kg/m^2^), mean (SD)	33.2 (5.7)	31.1 (5.3)	36.3 (6.5)	33.4 (5.6)	32.9 (5.8)
Non-black (%)^a^	99.3	99.4	99.4	99.1	99.3
Years since T2D diagnosis, mean (SD)	9.9 (7.0)	9.8 (7.0)	9.8 (6.7)	10.0 (6.7)	9.9 (6.9)
HbA1c (%), mean (SD)	8.3 (1.4)	8.0 (1.3)	8.4 (1.2)	8.6 (1.5)	8.3 (1.4)
HbA1c ≥ 8.5% (%)	32.6	23.2	34.3	36.5	30.7
Missing HbA1c (%)	9.9	6.6	5.0	8.0	8.3

(B) Clinical parameters relevant to CVD					
Systolic BP (mmHg), mean (SD)	137.3 (15.5)	136.7 (15.5)	136.6 (14.2)	136.7 (14.6)	137.0 (15.3)
Diastolic BP (mmHg), mean (SD)	81.8 (9.0)	80.8 (9.0)	81.3 (9.5)	82.3 (8.8)	81.6 (9.0)
Missing (%)	5.5	4.0	7.5	8.2	5.7
LVEF (%), mean (SD)	57.6 (10.2)	59.0 (9.7)	59.5 (9.0)	58.1 (8.1)	58.1 (9.9)
LVEF confirmed by echocardiography (%)	23.3	15.6	14.1	9.6	18.3
Missing (%)	76.1	84.2	85.3	89.6	81.2
Unknown (%)	0.5	0.3	0.3	0.3	0.4
Total cholesterol (mml/L), mean (SD)	5.0 (1.2)	5.0 (1.2)	4.9 (1.2)	5.1 (1.3)	5.0 (1.2)
Missing (%)	29.1	27.1	30.7	35.3	29.6
LDL (mml/L), mean (SD)	2.7 (1.0)	2.8 (1.1)	2.6 (1.0)	2.7 (1.0)	2.7 (1.0)
Missing (%)	44.8	44.1	41.0	51.0	45.1
HDL (mml/L), mean (SD)	1.2 (0.4)	1.2 (0.3)	1.2 (0.3)	1.2 (0.3)	1.2 (0.4)
Missing (%)	46.5	45.5	41.6	51.7	46.5
Triglycerides (mml/L), mean (SD)	1.9 (0.8)	1.9 (0.7)	2.2 (1.7)	2.0 (1.0)	2.0 (0.9)
Missing (%)	38.6	38.8	36.3	45.9	39.5

(C) Clinical parameters relevant to CKD					
Serum creatinine (mmol/L), mean (SD)	82.9 (21.4)	92.6 (33.0)	79.3 (19.2)	78.8 (16.8)	84.8 (25.1)
eGFR (ml/min), mean (SD)	78.2 (18.4)	69.5 (22.6)	84.1 (19.1)	81.3 (17.2)	76.7 (20.1)
Missing (%)	20.7	21.7	26.9	25.7	22.2
UACR (mg/g), mean (SD)	84.6 (123.6)	100.9 (241.9)	45.0 (94.2)	89.7 (134.3)	86.2 (107.4)
UACR (mg/g), median (Q1, Q3)	20.0 (4.6, 125.0)	12.7 (2.0, 123.0)	4.4 (0.9, 28.0)	23.5 (6.2, 96.0)	15.0 (2.7, 109.1)
Missing (%)	80.4	76.0	74.8	83.2	79.1

*BP* blood pressure, *BMI* body mass index, *CKD* chronic kidney disease, *CVD* cardiovascular disease, *DPP4i* dipeptidyl peptidase-4 inhibitor, *eGFR* estimated glomerular filtration rate, *Empa* empagliflozin, *GLP-1 RA* glucagon-like peptide-1 receptor agonist, *HbA1c* glycated haemoglobin, *HDL* high density lipoproteins, *LDL* low density lipoproteins, *LVEF* left ventricular ejection fraction, *Q* quartile, *SD* standard deviation, *SGLT2i* sodium-glucose cotransporter-2 inhibitor, *T2D* type 2 diabetes, *UACR* urine albumin-creatinine ratio.

^a^Assessment of ethnicity (black or non-black) was necessary for calculation of eGFR (shown in Table 1c).

**Table 2:** Patient characteristics at baseline by HCP specialty (Prescribed Patient Set)

HCP specialty	Endocrinologist				Diabetologist					Cardiologist					
Study medication and characteristic	Empa N = 815	DPP4i N = 504	GLP-1 RA N = 89	Other SGLT2i N = 244	Total N = 1652	Empa N = 1073	DPP4i N = 624	GLP-1 RA N = 271	Other SGLT2i N = 333	Total N = 2301	Empa N = 78	DPP4i N = 16	GLP-1 RA N = 1	Other SGLT2i N = 7	Total N = 102
(A) Demographics and clinical parameters relevant to T2D															
Age (years), mean (SD)	61.5 (9.7)	65.3 (10.4)	55.8 (10.5)	62.0 (9.2)	62.4 (10.2)	63.0 (9.4)	67.2 (11.0)	59.2 (10.8)	62.3 (9.2)	63.6 (10.3)	64.4 (8.4)	70.9 (9.5)	54.0	65.0 (9.4)	65.3 (8.9)
Female (%)	53.0	66.3	41.6	57.4	57.1	40.4	47.6	49.8	42.0	43.7	44.9	50.0	0	28.6	44.1
BMI (kg/m^2^), mean (SD)	33.3 (5.4)	31.3 (5.2)	36.7 (5.9)	34.3 (5.8)	33.0 (5.6)	33.3 (5.9)	31.0 (5.4)	36.2 (6.7)	32.8 (5.3)	33.0 (6.0)	30.8 (4.6)	29.9 (4.2)	25.8	30.7 (2.3)	30.6 (4.4)
Non-black (%)^a^	98.8	98.6	98.9	98.0	98.6	99.7	100	99.6	100	99.8	100	100	100	100	100
Years since T2D diagnosis, mean (SD)	9.1 (6.6)	9.1 (6.6)	9.2 (6.9)	9.8 (6.7)	9.2 (6.6)	10.7 (7.3)	10.4 (7.3)	10.1 (6.6)	10.1 (6.8)	10.5 (7.2)	6.7 (4.0)	9.7 (5.7)	6.0	10.9 (6.5)	7.5 (4.7)
HbA1c (%), mean (SD)	8.5 (1.4)	8.1 (1.2)	8.2 (1.1)	8.5 (1.3)	8.4 (1.3)	8.2 (1.3)	7.9 (1.4)	8.4 (1.2)	8.6 (1.6)	8.2 (1.4)	7.9 (1.1)	8.1 (1.1)	7.4	8.5 (0.7)	8.0 (1.1)
HbA1c ≥ 8.5% (%)	42.8	27.4	32.6	39.8	37.1	25.9	19.7	35.1	34.2	26.5	17.9	25.0	0	28.6	19.6
Missing HbA1c (%)	1.6	5.4	4.5	3.7	3.2	14.3	7.7	5.2	10.8	10.9	35.9	6.3	0	28.6	30.4

(B) Clinical parameters relevant to CVD															
Systolic BP (mmHg), mean (SD)	136.8 (14.4)	134.3 (13.5)	134.5 (12.7)	135.6 (14.4)	135.7 (14.1)	137.5 (16.2)	138.6 (16.5)	137.2 (14.5)	137.9 (14.6)	137.8 (15.9)	140.6 (16.8)	140.4 (24.4)	165.0	120.4 (10.4)	139.6 (18.7)
Diastolic BP (mmHg), mean (SD)	83.5 (7.8)	81.6 (7.8)	83.7 (8.5)	82.6 (8.2)	82.8 (8.0)	80.4 (9.5)	80.1 (9.8)	80.3 (9.6)	82.3 (9.0)	80.5 (9.5)	83.7 (10.8)	82.5 (13.0)	110.0	69.8 (10.0)	82.9 (11.9)
Missing (%)	1.6	0.2	0	2.5	1.2	7.3	7.1	10.0	12.0	8.2	23.1	6.3	0	28.6	20.6
LVEF (%), mean (SD)	59.5 (9.0)	61.7 (8.7)	63.2 (8.7)	58.1 (8.4)	59.9 (8.9)	54.0 (10.6)	59.3 (7.5)	57.7 (7.1)	59.8 (6.9)	56.6 (9.4)	56.0 (13.0)	43.8 (12.5)	32.0	48.3 (5.8)	52.8 (13.7)
LVEF confirmed by echocardiography (%)	35.1	15.5	24.7	16.4	25.8	11.2	13.6	10.3	3.9	10.7	66.7	93.8	100	42.9	69.6
Missing (%)	64.5	84.5	75.3	83.2	74.0	88.1	85.9	88.9	94.9	88.6	33.3	6.3	0	57.1	30.4
Unknown (%)	0.4	0	0	0	0	0.6	0.5	0.4	0.6	0.5	0	0	0	0	0
Total cholesterol (mml/L), mean (SD)	5.4 (1.2)	5.4 (1.2)	5.4 (1.3)	5.6 (1.2)	5.4 (1.2)	4.6 (1.1)	4.6 (1.1)	4.7 (1.2)	4.6 (1.1)	4.6 (1.1)	4.9 (1.3)	4.0 (1.1)	5.1	3.4 (0.3)	4.7 (1.3)
Missing (%)	19.3	16.3	23.6	21.7	18.9	36.6	36.1	33.2	45.3	37.3	29.5	18.8	0	28.6	27.5
LDL (mml/L), mean (SD)	2.9 (1.0)	3.1 (1.1)	3.0 (1.1)	3.0 (1.1)	3.0 (1.1)	2.5 (1.0)	2.5 (0.9)	2.5 (0.9)	2.6 (0.9)	2.5 (1.0)	2.7 (1.0)	2.3 (1.0)	2.1	1.8 (0.2)	2.5 (1.0)
Missing (%)	40.9	44.4	40.4	54.1	43.9	47.7	44.4	41.3	49.2	46.3	44.9	18.8	0	28.6	39.2
HDL (mml/L), mean (SD)	1.3 (0.4)	1.2 (0.3)	1.3 (0.4)	1.2 (0.3)	1.3 (0.4)	1.2 (0.3)	1.2 (0.3)	1.1 (0.3)	1.2 (0.3)	1.2 (0.3)	1.2 (0.3)	1.2 (0.4)	1.0	0.9 (0.2)	1.2 (0.3)
Missing (%)	45.3	48.6	43.8	57.4	48.0	47.7	43.4	41.0	48.0	45.8	43.6	25.0	0	28.6	39.2
Triglycerides (mml/L), mean (SD)	2.0 (0.8)	1.9 (0.7)	2.0 (0.6)	2.1 (1.1)	2.0 (0.8)	1.9 (0.8)	1.8 (0.7)	2.2 (1.9)	2.0 (0.9)	1.9 (1.0)	1.8(0.7)	1.8(0.9)	3.0	2.0(0.5)	1.8(0.7)
Missing (%)	32.3	37.7	38.2	44.3	36.0	43.3	40.2	35.8	47.4	42.2	38.5	18.8	0	28.6	34.3

(C) Clinical parameters relevant to CKD															
Serum creatinine (mmol/L), mean (SD)	83.9 (16.1)	92.2 (28.0)	82.6 (17.9)	79.3 (14.5)	85.7 (20.8)	82.0 (25.4)	92.9 (37.4)	78.1 (19.7)	78.7 (18.7)	84.1 (28.5)	82.3 (23.4)	94.6 (34.2)	67.0	60.4 (3.6)	82.8 (25.6)
eGFR (ml/min), mean (SD)	75.9 (17.0)	67.7 (20.8)	84.3 (19.6)	78.8 (17.0)	74.3 (19.0)	80.4 (19.3)	71.5 (24.1)	84.0 (18.9)	83.3 (17.1)	78.8 (20.9)	78.7 (19.1)	67.2 (24.0)	103.6	101.1 (4.8)	78.5 (20.7)
Missing (%)	8.5	11.3	15.7	12.7	10.4	29.7	30.1	30.6	35.1	30.7	24.4	18.8	0	28.6	23.5
UACR (mg/g), mean (SD)	129.1 (139.5)	133.0 (137.5)	108.2 (131.0)	112.7 (137.2)	126.4 (137.8)	20.2 (38.7)	74.0 (300.8)	18.3 (56.3)	44.5 (117.8)	41.1 (187.3)	298.6 (133.8)	–	–	–	298.6 (133.8)
UACR (mg/g), median (Q1, Q3)	68.0 (15.0, 235.0)	70.5 (10.0, 213.0)	45.0 (10.0, 203.0)	37.0 (12.3, 190.0)	69.3 (12.0, 223.0)	7.0 (1.6, 18.0)	4.8 (1.2, 17.8)	1.7 (0.6, 6.4)	7.0 (2.0, 32.9)	4.9 (1.2, 17.8)	282.0 (222.0, 405.0)	–	–	–	282.0 (222.0, 405.0)
Missing (%)	74.6	75.2	70.0	73.4	74.3	84.2	76.1	76.4	90.1	81.9	89.7	100	100	100	92.2

*BP* blood pressure, *BMI* body mass index, *CKD* chronic kidney disease, *CVD* cardiovascular disease, *DPP4i* dipeptidyl peptidase-4 inhibitor, *eGFR* estimated glomerular filtration rate, *Empa* empagliflozin, *GLP-1 RA* glucagon-like peptide-1 receptor agonist, *HbA1c* glycated haemoglobin, *HCP* healthcare professional, *HDL* high density lipoproteins, *LDL* low density lipoproteins, *LVEF* left ventricular ejection fraction, *Q* quartile, *SD* standard deviation, *SGLT2i* sodium-glucose cotransporter-2 inhibitor, *T2D* type 2 diabetes, *UACR* urine albumin-creatinine ratio.

^a^Assessment of ethnicity (black or non-black) was necessary for calculation of eGFR (shown in Table 2c

Systolic blood pressure and blood lipid levels tended to be comparable per T2D study medication in the overall population (Table 1a) and in each HCP specialty subgroup (Table 2b). In the overall population, patients treated with DPP4i had marginally lower mean diastolic blood pressure than patients treated with the other study medications (80.8 mmHg vs 81.3–82.3 mmHg) and patients treated with GLP-1 RA had marginally higher triglyceride levels (2.2 mml/L vs 1.9–2.0) (both p < 0.01) (Table 1b). Comparisons of left ventricular ejection fraction (LVEF) were hampered by a large amount of missing data (81.2% of patients did not have LVEF values).

In the overall population, mean eGFR levels were lowest in the DPP4i group (69.5 ml/min) and highest in the GLP-1 RA group (84.1 ml/min) (p < 0.0001) (Table 1c). This was also the case per HCP specialty (Table 2c). Patients treated by endocrinologists had higher mean urine albumin-creatinine ratio (UACR) (range, 108.2–133.0 mg/g across the treatment groups) than patients treated by diabetologists (18.3–74.0 mg/g). These mean values were substantially higher than median UACR (37.0–70.5 mg/g and 1.7–7.0 mg/g, respectively) and comparisons of UACR were hampered by a large amount of missing data (74.3–92.2% of patients, across the HCP specialties, did not have UACR values).

### Secondary outcomes: reasons for choosing T2D study medication

**Fig. 3:**
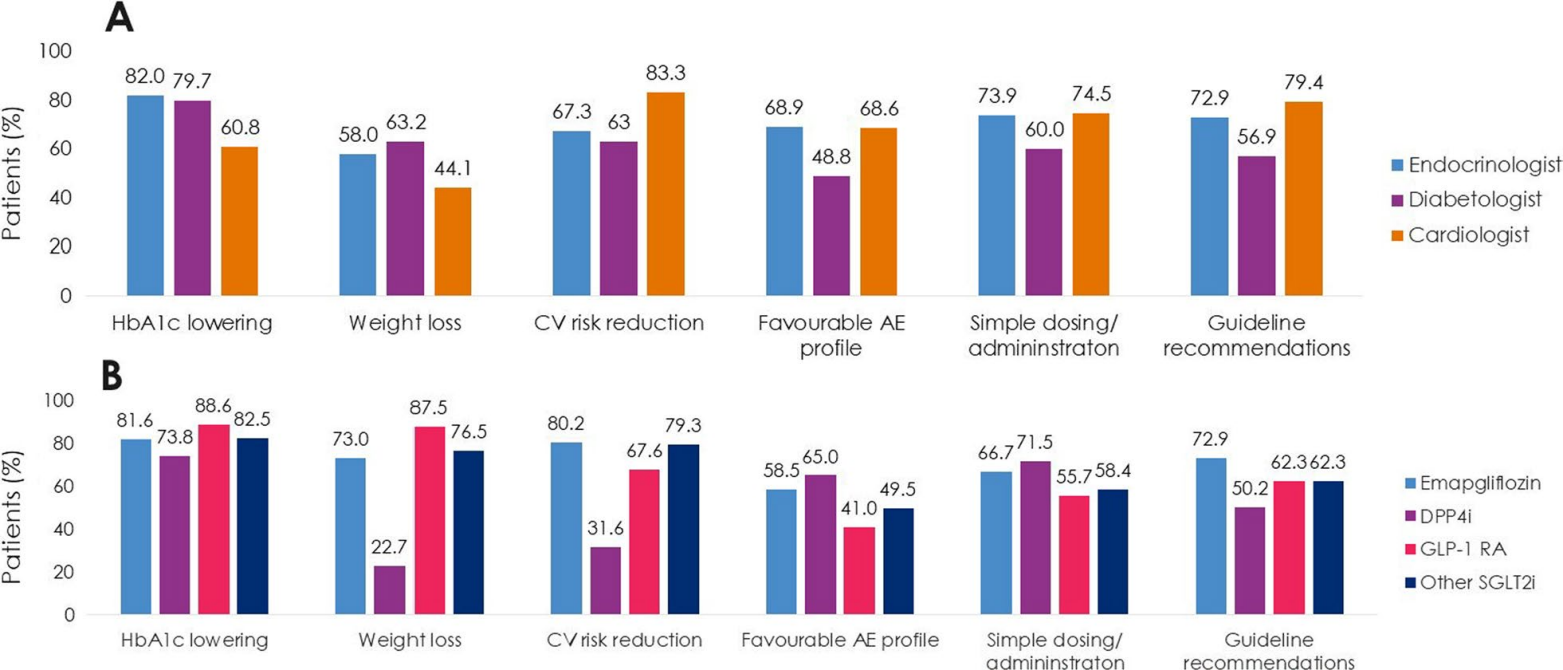
‘Highly relevant’ reasons for choosing T2D study medications **A** by HCP specialty **B** by treatment (Prescribed Patient Set). AE, adverse event; CV, cardiovascular; DPP4i, dipeptidyl peptidase-4 inhibitor; GLP-1 RA, glucagon-like peptide-1 receptor agonist; HbA1c, glycated haemoglobin; HCP, healthcare professional; SGLT2i, sodium-glucose cotransporter-2 inhibitor; T2D, type 2 diabetes

‘Guideline recommendations’ were chosen as a highly relevant reason to prescribe T2D medications for greater proportions of endocrinologists (72.9%) and cardiologists (79.4%) than diabetologists (56.9%) (Fig. 3). ‘HbA1c lowering’ was highly relevant for greater proportions of endocrinologists (82.0%) and diabetologists (79.7%) than cardiologists (60.8%). ‘CV risk reduction’ was highly relevant for a greater proportion of cardiologists (83.3%) than endocrinologists (67.3%) and diabetologists (63.0%).

### Secondary outcomes: other physicians involved in the choice of T2D study medications

For 88.1% of patients (diabetologists 90.7%, endocrinologists 86.0%, cardiologists 63.7%), HCP specialists did not involve other physicians in the choice of study medication. When consultations did occur, endocrinologists mainly involved cardiologists (8.0% of all endocrinologist-treated patients), other endocrinologists (6.3%), or general practitioners (GPs) (5.9%). Diabetologists mainly involved other diabetologists (6.3%). Cardiologists mainly involved endocrinologists (18.6%) or diabetologists (10.8%).

### Secondary outcomes: concomitant T2D, CVD, and CKD medications at baseline

For each HCP specialty, about 80% of patients received metformin (Table 3). Diabetologists had the highest percentage of patients receiving insulin (28.6%), compared with endocrinologists (20.7%) and cardiologists (16.7%). As expected, patients treated by cardiologists were more likely to receive various concomitant CVD and/or CKD medications than those treated by endocrinologists and diabetologists.

**Table 3:** Concomitant T2D and CVD/CKD medications at baseline (Prescribed Patient Set)

T2D medication (%)	Endocrinologist N = 1652	Diabetologist N = 2301	Cardiologist N = 102	All HCP specialties N = 4055
Metformin	81.7	78.0	79.4	79.5
Sulfonylurea	37.9	22.3	16.7	28.5
Acarbose	0.2	2.1	2.9	1.4
Pioglitazone	0.2	4.0	0	2.3
Insulin	20.7	28.6	16.7	25.1
Others	3.6	4.0	6.9	3.9
CVD/CKD medication (%)				
Antihypertensives (ACEi/ARB)	76.0	66.8	85.3	71.0
Statins	59.5	57.7	80.4	59.0
Low dose aspirin	33.6	27.0	62.7	30.6
β-blockers	35.1	40.1	71.6	38.8
Diuretics	28.5	31.8	43.1	30.8
Others	9.7	19.8	28.4	15.9

*ACEi* angiotensin converting enzyme inhibitors, *ARB* angiotensin receptor blockers, *CKD* chronic kidney disease, *CVD* cardiovascular disease, *HCP* healthcare professional, *T2D* type 2 diabetes

### Secondary outcomes: prevalence of CVD, CKD, and associated risk factors at baseline

**Fig. 4:**
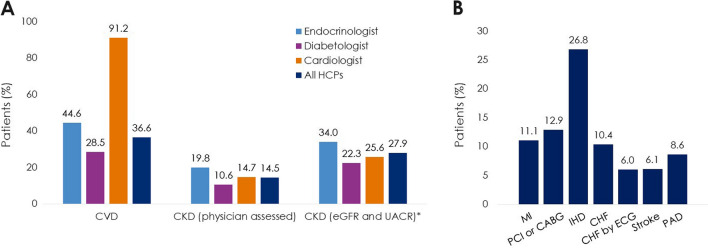
Prevalence of **A** CVD and CKD **B** types of CVD at baseline (Prescribed Patient Set). *These laboratory values were available for 78.3% (n = 3175) of 4055 patients in the Prescribed Patient Set. Thus, the denominators used to calculate the percentages of patients with CKD according to eGFR and UACR were 3175 (all HCPs), 1493 (endocrinologist group), 1604 (diabetologist group), 78 (cardiologist group). CABG, coronary artery bypass graft; CHF, congestive heart failure; CHF by ECG, CHF confirmed by electrocardiography; CKD, chronic kidney disease. CVD, cardiovascular disease; DPP4i, dipeptidyl peptidase-4 inhibitor; eGFR, estimated glomerular filtration rate; GLP-1 RA, glucagon-like peptide-1 receptor agonist; HCP, healthcare professional; IHD, ischaemic heart disease; MI, myocardial infarction; PAD, peripheral arterial disease; PCI, percutaneous coronary intervention; SGLT2i, sodium-glucose cotransporter-2 inhibitor; UACR, urine albumin-creatinine ratio

Overall, 36.6% of patients had diagnosed CVD (Fig. 4a). The most prevalent CVD subtype was ischaemic heart disease (26.8%) (Fig. 4b). The prevalence of CKD depended on whether it was based on HCP assessment (14.5%) or on eGFR and UACR laboratory values (27.9%) (Fig. 4a). Cardiologists had the highest proportion of patients with CVD (91.2%) (Fig. 4a). Endocrinologists had the second highest proportion of patients with CVD (44.6%), and highest with CKD (physician assessed, 19.8%; eGFR and UACR status, 34.0%).

Regarding risk factors for CVD and CKD, endocrinologists and diabetologists had similar distributions of patients who were overweight, obese, and hypertensive (Table 4). Endocrinologists and cardiologists treated greater percentages of patients with a family history of early onset cardiorenal diseases than diabetologists, although many patients had unknown family history. As expected, patients treated by cardiologists had a higher mean 10-year fatal CVD risk than patients treated by endocrinologists or diabetologists. In the endocrinologist subgroup, patients receiving empagliflozin had a higher mean 10-year fatal CVD risk than patients prescribed the other three medications (p = 0.013) and, in the diabetologist subgroup, patients receiving empagliflozin or DPP4i had a higher mean 10-year fatal CVD risk than patients prescribed GLP-1 RA or other SGLT2i (p = 0.003) (Table 4).

**Table 4:** CVD and CKD risk factors at baseline (Prescribed Patient Set)

HCP specialty	Endocrinologist					Diabetologist					Cardiologist				
Study medication and characteristic	Empa N = 815	DPP4i N = 504	GLP-1 RA N = 89	Other SGLT2i N = 244	Total N = 1652	Empa N = 1073	DPP4i N = 624	GLP-1 RA N = 271	Other SGLT2i N = 333	Total N = 2301	Empa N = 78	DPP4i N = 16	GLP-1 RA N = 1	Other SGLT2i N = 7	Total N = 102
(A) Categorically assessed CVD and CKD risk factors															
BMI (%)															
Overweight	25.6	36.5	11.2	18.9	27.2	23.4	37.2	10.7	25.5	25.9	37.2	43.8	100	42.9	39.2
Obese	72.0	56.0	87.6	78.3	68.9	72.2	51.9	86.3	68.8	67.9	55.1	50	0	57.1	53.9
Hypertension (%)	87.5	83.7	85.4	83.2	85.6	85.6	80.0	85.6	82.6	83.7	89.7	100	100	85.7	91.2
Unknown	0.4	0.2	0	0.4	0.3	1.1	1.6	1.8	0.6	1.3	0	0	0	0	0
Never smoked (%)	52.9	66.3	52.8	58.2	57.7	43.7	54.2	56.1	48.0	48.6	46.2	50	100	71.4	49.0
Unknown	4.8	4.8	4.5	7.0	5.1	13.4	9.6	6.3	8.4	10.8	1.3	6.3	0	0	2.0
Physically inactive (%)*	57.4	52.6	59.6	50.4	55.0	43.1	48.1	50.6	54.7	47	50.0	93.8	100	57.1	56.9
Unknown	5.0	10.1	4.5	9.4	7.2	7.5	6.9	5.9	3.3	6.5	1.2	0	0	14.3	2.0
Family history (%)															
Early onset heart disease	37.6	29.4	39.3	32	34.3	25.7	18.1	23.3	22.2	22.9	46.2	18.8	0	0	38.2
Unknown	18.5	27.2	18.0	22.1	21.7	27.0	23.7	25.5	31.8	26.6	6.4	6.3	0	14.3	6.9
Early onset kidney disease	8.1	7.3	4.5	4.9	7.2	6.9	4.2	4.8	7.2	6.0	11.5	6.3	0	0	9.8
Unknown	22.8	31.9	18.0	27.9	26.1	34.6	27.4	27.3	36.9	32.1	10.3	6.3	0	14.3	9.8

(B) Ten-year fatal CVD risk (SCORE Risk Chart)															
10 year fatal CVD risk, mean (SD)	6.4 (5.7)	5.6 (4.7)	4.3 (3.7)	6.0 (5.7)	6.0 (5.3)	7.0 (5.5)	7.0 (5.9)	5.4 (4.7)	6.5 (5.1)	6.7 (5.5)	8.9 (7.7)	9.4 (7.8)	5.0	3.8 (2.3)	8.6 (7.5)
Missing (%)	24.5	20.0	30.3	26.6	23.8	43.2	43.1	42.8	50.8	44.2	30.8	31.3	0	28.6	30.4

*BMI* body mass index, *CKD* chronic kidney disease, *CVD* cardiovascular disease, *DPP4i* dipeptidyl peptidase-4 inhibitor, *Empa* empagliflozin, *GLP-1 RA* glucagon-like peptide-1 receptor agonist, *HCP* healthcare professional, *SD* standard deviation, *SGLT2i* sodium-glucose cotransporter-2 inhibitor, *T2D* type 2 diabetes

*Less than 2.5 h of moderate or 75 min of vigorous aerobic exercise per week

### Secondary outcomes: treatment use in patients with and without CVD at baseline

**Fig. 5:**
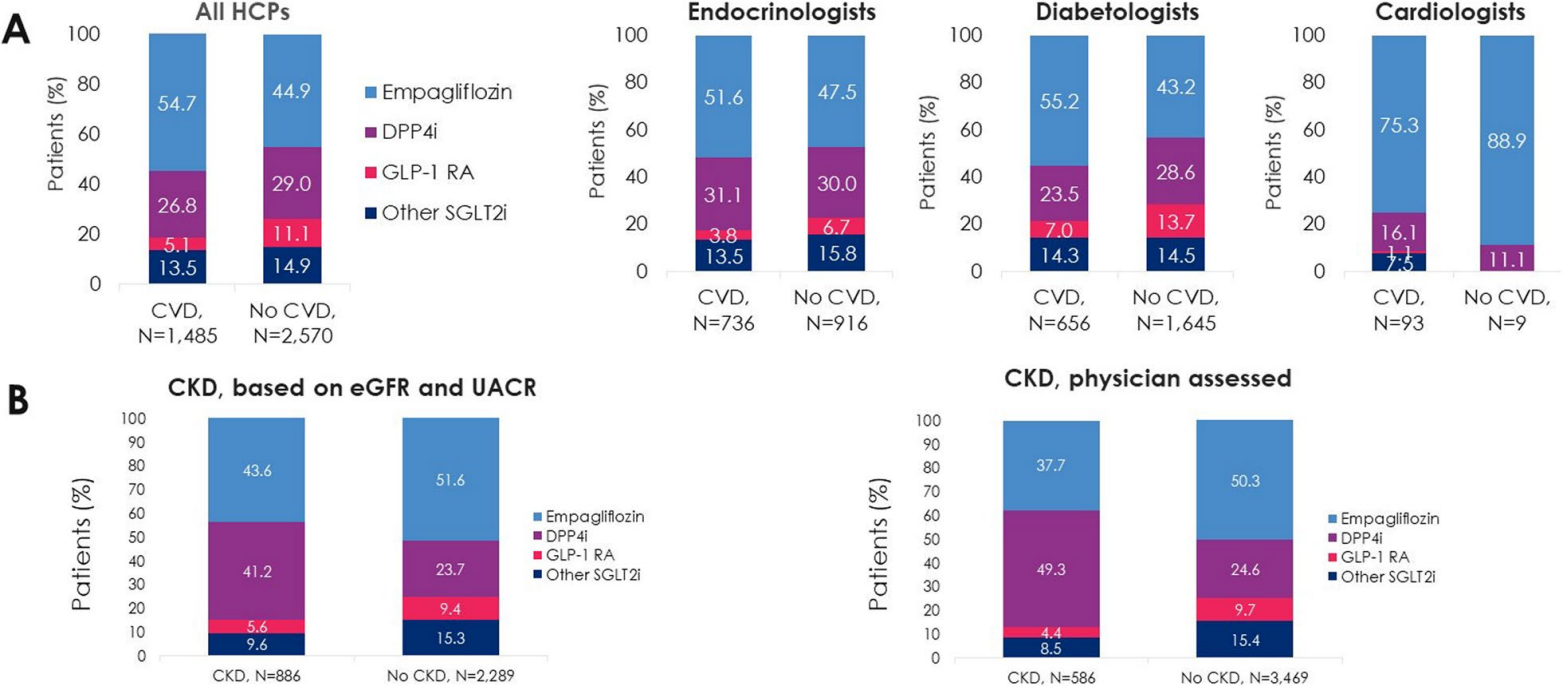
T2D study medication use in patients with and without **A** CVD and **B** CKD at baseline (Prescribed Patient Set). CVD, cardiovascular disease; CKD, chronic kidney disease; DPP4i, dipeptidyl peptidase-4 inhibitor; GLP-1 RA, glucagon-like peptide-1 receptor agonist; HCP, healthcare professional; SGLT2i, sodium-glucose cotransporter-2 inhibitor

Overall, 73.2% of patients with CVD were prescribed an SGLT2i or GLP-1 RA; the remainder (26.8%) received DPP4i (Fig. 5a). When analysed by HCP specialty, this treatment pattern was also seen in patients treated by endocrinologists and by diabetologists (31.1% and 23.5% of patients with CVD, respectively, received DPP4i).

Empagliflozin was the most used T2D medication, both for patients with CVD (54.7%) and without CVD (44.9%) (Fig. 5a), and for all types of CVD (61.3–66.9% of patients with myocardial infarction, cardiology intervention [PCI or CABG], or congestive heart failure confirmed by echocardiography, and 48.9–59.9% of patients with ischaemic heart disease, congestive heart failure, peripheral arterial disease, or stroke) (data not shown in the Figures). The least used medication was GLP-1 RA, both for patients with CVD (5.1%) and without CVD (11.1%) (Fig. 5a).

### Additional analysis: treatment use in patients with and without CKD at baseline

Despite differences in prevalence of CKD (Fig. 4a), treatment patterns were comparable based on physician and laboratory assessments (Fig. 5b). According to laboratory assessments, most patients with CKD received DPP4i (41.2%) or empagliflozin (43.6%), and most patients without CKD received empagliflozin (51.6%) or DPP4i (23.7%) (Fig. 5b).

### Secondary outcomes: associations between socioeconomics and treatment decisions at baseline

Employment was lowest in the DPP4i group (37.8%), compared with empagliflozin (49.1%), other SGLT2i (51.7%), and GLP-1 RA (63.4%). This was consistent with mean ages: 66.4 years (SD 10.8) in the DPP4i group, 62.4 years (SD 9.5) in the empagliflozin group, 62.2 years (SD 9.2) in the other SGLT2i group, and 58.3 years (SD 10.8) in the GLP-1 RA group.

Across the treatment groups, 87.7–93.1% of patients had statutory insurance, and 2.7–4.5% had private insurance; empagliflozin had the lowest statutory (87.7%) and highest private (4.5%) insurance rates. Endocrinologists had the highest percentage of privately insured patients (8.8%), compared with cardiologists (3.9%) and diabetologists (0.2%), i.e. 94.8% of privately insured patients were treated by endocrinologists. In the endocrinologist subgroup, 14.6% of patients who received GLP-1 RA were privately insured, compared with 10.1% for empagliflozin, 6.7% for DPP4i, and 6.6% for other SGLT2i.

### Secondary outcomes: discontinuations at 1 year ± 2 months of treatment

**Fig. 6:**
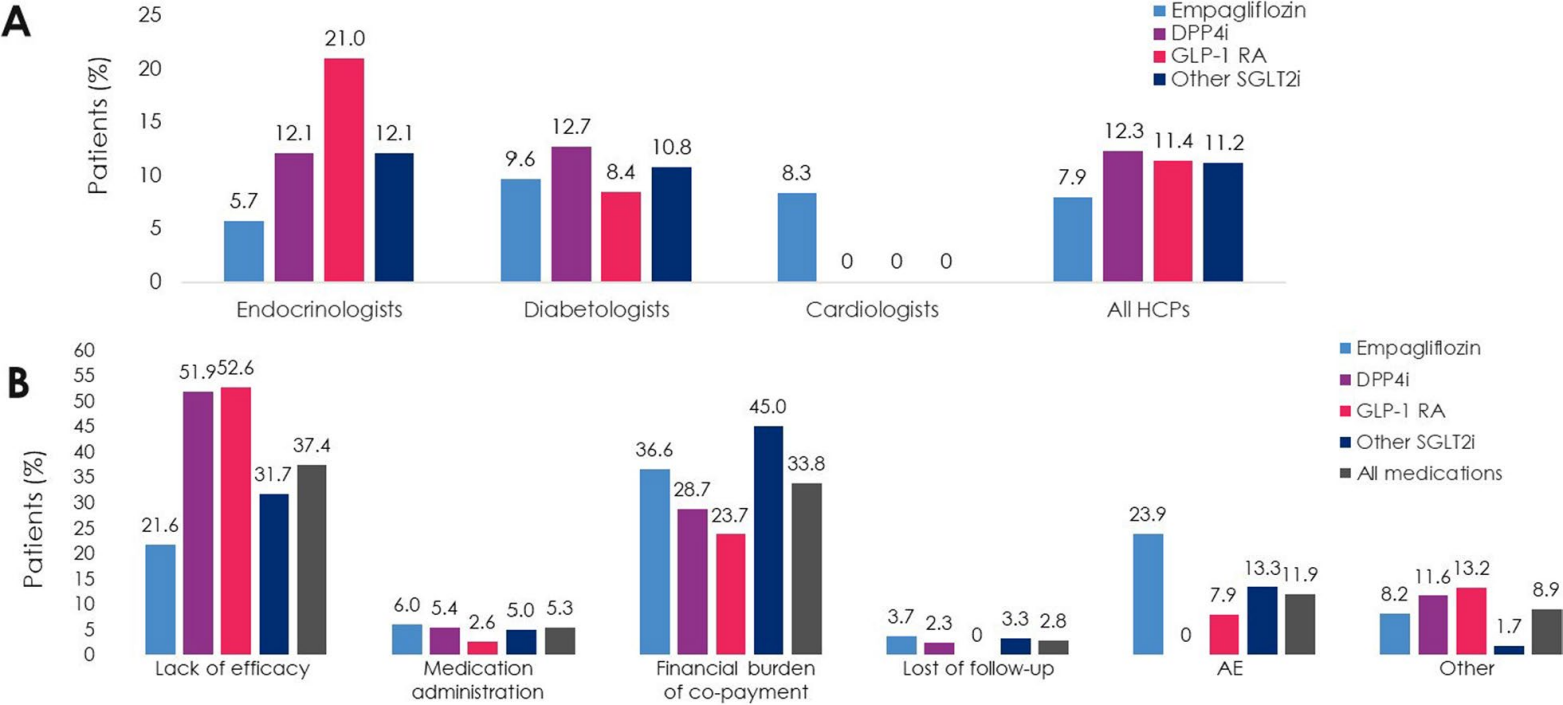
**A** Discontinuations at 1 year ± 2 months of treatment and **B** primary reasons for discontinuing T2D study medications (Full Analysis Set). AE, adverse event; DPP4i, dipeptidyl peptidase-4 inhibitor; GLP-1 RA, glucagon-like peptide-1 receptor agonist; HCP, healthcare professional; SGLT2i, sodium-glucose cotransporter-2 inhibitor; T2D, type 2 diabetes

Overall, 10.0% of patients had discontinued T2D study medication 1 year ± 2 months after initiating treatment (range, 7.9% for empagliflozin, 12.3% for DPP4i; Fig. 6a). The mean time to discontinuation was 19.8 months (standard error [SE] 0.4) for all study medications and, in ascending order, 14.0 months (SE 0.1) for other SGLT2i, 18.3 months (SE 0.4) for DPP4i, 19.5 months (SE 0.7) for empagliflozin, and 20.6 months (SE 0.6) for GLP-1 RA (data not shown in the Figures).

Reimbursement (‘financial burden of co-payment’) was the most common primary reason for discontinuing empagliflozin (36.6%) or another SGLT2i (45.0%), and the second most common for DPP4i (28.7%) and GLP-1 RA (23.7%) (Fig. 6b). Patients discontinued DPP4i or GLP-1 RA primarily due to lack of efficacy (51.9% and 52.6%, respectively), compared with empagliflozin (21.6%) and other SGLT2i (31.7%). None of 1053 patients discontinued DPP4i because of an AE (Fig. 6b and Table 5), while 23.9%, 13.3%, and 7.9% of patients who discontinued empagliflozin, another SGLT2i, or GLP-1 RA, respectively, did so due to an AE (Fig. 6b); the actual proportions of patients who discontinued due to an AE were 1.9% of all patients treated with empagliflozin (n = 32, N = 1697), 1.5% of all patients treated with another SGLT2i (n = 8, N = 536), and 0.9% of all patients treated with GLP-1 RA (n = 3, N = 332) (Table 5). In the overall population, only four types of AE led to discontinuation of study medication by five or more patients: dysuria (0.30%; n = 11), balanitis and other genital infections (0.19%; n = 7), urinary tract infection (0.14%; n = 5), and vulvovaginitis (0.14%; n = 5). One CV AE (a cerebrovascular event) led to discontinuation of empagliflozin (Table 5).

**Table 5:** Adverse events leading to discontinuations of T2D study medication at 1-year ± 2-months of treatment (Full Analysis Set)

AE leading to discontinuation, n (%)	Empagliflozin (N = 1697)	GLP-1 RA (N = 332)	Other SGLT2i (N = 536)	Total (N = 3618)
Total AEs	32 (1.89)	3 (0.90)	8 (1.49)	43 (1.19)
Gastrointestinal AEs				
Nausea	1 (0.06)	2 (0.60)		3 (0.08)
Dyspepsia	2 (0.12)			2 (0.06)
Vomiting	2 (0.12)			2 (0.06)
Abdominal pain	3 (0.18)			3 (0.08)
Other		1 (0.30)		1 (0.03)
Genitourinary infections				
Vaginal moniliasis			1 (0.19)	1 (0.03)
Vulvovaginitis	4 (0.24)		1 (0.19)	5 (0.14)
Balanitis and other genital infections	6 (0.35)		1 (0.19)	7 (0.19)
Urinary tract infection (including pyelonephritis and urosepsis)	4 (0.24)		1 (0.19)	5 (0.14)
Renal AEs				
Glomerular filtration rate decreased	1 (0.06)			1 (0.03)
Urinary AEs				
Increased urination	3 (0.18)		1 (0.19)	4 (0.11)
Dysuria	9 (0.53)		2 (0.37)	11 (0.30)
Other	1 (0.06)		1 (0.19)	2 (0.06)
Metabolic AEs				
Thirst	1 (0.06)			1 (0.03)
Cardiovascular AEs				
Cerebrovascular event	1 (0.06)			1 (0.03)

*AE* adverse event, *DPP4i* dipeptidyl peptidase-4 inhibitor, *GLP-1 RA* glucagon-like peptide-1 receptor agonist, *SGLT2i* sodium-glucose cotransporter-2 inhibitor

For 79.5% of discontinuations (diabetologists 86.9%, endocrinologists 71.3%), no other physician was involved in the decision. When other physicians were involved, they were mainly GPs (10.5% of all discontinuations), although endocrinologists primarily involved other endocrinologists (18.2%) ahead of GPs (9.8%).

## Discussion

The CORDIALLY real-world study was conducted to improve insight into the treatment patterns of patients with T2D initiating modern antidiabetic medications (SGLT2i, GLP-1 RA, and DPP4i) in five CEE countries (Bulgaria, Czech Republic, Hungary, Poland, and Russia), based on a snapshot taken in autumn 2018. Notable findings include that empagliflozin, received by 48.5% of patients, was the most prescribed T2D study medication by all three HCP specialties (endocrinologists, diabetologists, cardiologists); more than three times the number of patients were prescribed empagliflozin than all other SGLT2i, and 10 times more among cardiologists. Overall, 26.8% of patients with T2D and comorbid CVD received DPP4i and not SGLT2i or GLP-1 RA, despite guideline recommendations at the time of prescription to use SGLT2i or GLP-1 RA due to CV benefits in this patient population.

In CORDIALLY, patients initiating DPP4i were older than those prescribed SGLT2i or GLP-1 RA, consistent with findings in the US and Denmark [30, 31]. For elderly patients, who likely present with various complications, physicians may consider DPP4i to be particularly safe and well tolerated, with a moderate effect on glucose lowering that may be sufficient when relaxed glucose targets apply. Treatment guidelines suggest that glycaemic targets may be relaxed for frail older adults [35]. Prescription of DPP4i, rather than SGLT2i or GLP-1 RA, may be related to perceived benefit-risk. Discussing potential AEs of treatment options with elderly patients (often with cognitive decline) may also be time consuming, and they may not understand and adequately act on instructions, potentially exposing them to unnecessary risk. Therefore, physicians may choose a quick ‘glucocentric’ solution and safety-first principle. However, CVOTs and other studies demonstrate that older patients benefit from treatment with SGLT2i or GLP-1 RA [5, 6, 9, 36, 37], without discernible differences in safety across the age groups for empagliflozin (< 65, 65–74, ≥ 75 years) [36] and dulaglutide (< 65, ≥ 65 years) [37]. For SGLT2i, AEs include rare but potentially serious diabetic ketoacidosis, and urogenital infections that tend to be mild-to-moderate and manageable [28, 38]. An interim analysis of the EMPRISE real-world study reported patients with T2D, with and without history of CVD, could benefit from an approximately 50% lower risk of hospitalisation for heart failure when treated with empagliflozin versus DPP4i [23]. In our opinion, the proven cardiorenal benefits of SGLT2i outweigh the risks of potential AEs, including favourable numbers needed to treat (39/3.1 years for death from any cause with empagliflozin [5]; 23/2.6 years for cardiorenal events with canagliflozin [39]) and, in EMPA-REG OUTCOME, statistically significantly lower risk of serious AEs with 10 mg or 25 mg daily empagliflozin (38.2%) than placebo (42.3%) [5]. In CORDIALLY, although no patients receiving DPP4i (N = 1053) discontinued this treatment due to an AE, discontinuation rates owing to an AE were low for empagliflozin (1.9%), other SGLT2i (1.5%), and GLP-1 RA (0.9%). AEs associated with discontinuation of SGLT2i were predominantly genitourinary infections and other urinary AEs, while the main AE leading to discontinuation of GLP-1 RA was nausea.

CORDIALLY also demonstrates that modern T2D medications can be administered in the long term, based on a discontinuation rate of 10.0% (range, 7.9–12.3%) and mean time to discontinuation of 19.8 months. Reimbursement (‘financial burden of co-payment’) was the most common primary reason for discontinuing empagliflozin (36.6%) and other SGLT2i (45.0%) and the second most common for DPP4i (28.7%) and GLP-1 RA (23.7%). At baseline in CORDIALLY (autumn 2018), the average price (including tax) of a daily dose of empagliflozin 10 mg (€38.19) was only 13% (€4.48) higher than for linagliptin 5 mg across the five CEE countries; when studies in the US and Denmark suggested preference for DPP4i over SGLT2i in patients who would derive greater benefit from SGLT2i treatment, the prices of the two classes were similar [28]. Another potential explanation for use of DPP4i, rather than recommendations to initiate SGLT2i and GLP-1 RA treatment in patients with T2D, relates to emphasis placed on guidelines; these were highly relevant for greater proportion of cardiologists (79.4%) and endocrinologists (72.9%) than diabetologists (56.9%). Leading diabetologists from the CEE region recently argued that HCPs’ preference for familiar treatments, lack of awareness, competing priorities from more apparent risks (e.g. hypoglycaemia and obesity), and lack of cooperation between HCP specialties may limit prescription of modern T2D medications [28]. In the current study, few HCPs consulted other HCPs on treatment choice and decisions to discontinue study medications.

Empagliflozin was the first T2D medication to demonstrate CV benefits in 2015 and, so far, EMPA-REG OUTCOME is the only CVOT of a modern T2D medication to report statistically significant reduction in both a composite of major adverse CV events and CV mortality in a cohort of patients with T2D and established CVD [5]. This is likely reflected by treatment patterns, with empagliflozin the most prescribed T2D medication in CORDIALLY (received by 48.5% of patients; 33.3–70.0% in each of the five CEE countries). Cardiologists had the highest percentage of patients with prescriptions of empagliflozin (76.5%), consistent with the high prevalence of CVD for patients treated by this HCP specialty (91.2%). However, in the overall population, empagliflozin was commonly prescribed for patients without CVD (44.9%), as well as with CVD (54.7%). HCPs’ reasons for choosing T2D medications, and baseline characteristics (e.g. concomitant treatments), reflect that cardiologists focus on treating CVDs while considering T2D, whereas T2D specialists focus on treating hyperglycaemia while considering CVDs.

In this real-world study, the prevalence of CKD depended on whether it was based on HCP assessment (14.5%) or on eGFR and UACR laboratory values (27.9%), suggesting under-diagnosis of CKD in patients with T2D. Regardless of assessment method, approximately half of the patients with CKD received prescriptions for empagliflozin or another SGLT2i in autumn 2018. Most of the remaining patients with CKD received DPP4i, despite accumulated evidence for renal benefits with SGLT2i [15–17, 40] that have not been demonstrated with DPP4i [41–44] and 2018 guideline recommendations to preferentially use SGLT2i (or, if not tolerated or contraindicated, GLP-1 RA with proven CVD benefit) when CKD predominates [27].

While CORDIALLY benefited from a sizable population (4055 eligible patients) across three HCP specialties and five CEE countries, there are several notable limitations. Only 102 patients (2.5%) were enrolled by cardiologists; this relatively low number of patients may be related to regulations that allow reimbursement of the new medications only when prescribed by endocrinologists and diabetologists. Regarding prescription of DPP4i for 26.8% of patients with T2D and comorbid CVD, despite recommendations to use SGLT2i or GLP-1 RA due to CV benefits, it is feasible that in some instances these medications were justifiably prescribed by physicians considering circumstances that were not detected in this study. Although reasons for choice of study medication were reported, these were limited to a prespecified list. Similarly, why empagliflozin was preferred over other SGLT2i was not directly captured by the study, although this is likely related to the aforementioned CV benefits of empagliflozin that were reported in 2015 [5]. Whether there are any clinically meaningful differences between empagliflozin and other SGLT2i is unknown. Some information (e.g. family history of early cardiorenal diseases) was unknown or missing from retrospectively assessed eCRFs, and only AEs related to discontinuation of study medication had to be recorded in the eCRFs. Some outcomes may have been affected by confounding factors (e.g. imbalanced patient enrolment per country, selection of *primary* reasons for discontinuation rather than being able to select more than one reason per patient). One potential confounding factor is that whether endocrinologists or diabetologists were the predominant specialty differed by country; however, many outcomes were similar between the endocrinologist and diabetologist groups. It is also notable that this study was based on patients initiating T2D medications in autumn 2018. If this study was repeated with current data, recent global developments (e.g. publication of new findings regarding treatment of patients with heart failure and CKD using SGLT2i [15, 18, 19]) and national developments (e.g. increased state reimbursement of SGLT2i in Russia) would in all likelihood affect the findings. We would also expect physicians to be more familiar with updated treatment guidelines and more confident regarding the effectiveness and safety of modern antidiabetic medications.

In summary, the CORDIALLY real-world study was conducted to improve insight into the baseline characteristics and treatment patterns of patients with T2D initiating modern antidiabetic medications in CEE countries. Based on data collected in autumn 2018, empagliflozin was the most prescribed study medication, received by 48.5% of patients across five CEE countries, and was the main study medication prescribed by all three HCP specialties (endocrinologists, diabetologists, cardiologists). Overall, 26.8% of patients with T2D and comorbid CVD received DPP4i but not SGLT2i or GLP-1 RA, despite guideline recommendations at the time of prescription to use SGLT2i or GLP-1 RA due to CV benefits in this patient population. Many patients with CKD (≥ 41%) received DPP4i, despite guidelines recommending SGLT2is owing to their renal benefits. Thus, significant numbers of patients in this real-world study, who initiated treatment for T2D in CEE countries in autumn 2018, met the criteria for early treatment with cardioprotective T2D medications but were not treated accordingly. The probable consequences include unnecessary CV events, heart failure, and premature deaths; hence, patients with T2D and comorbid CVD/CKD or high CV risk require greater access to modern antidiabetic medications to gain from their non-glycaemic benefits. These findings should be discussed and addressed by clinicians and health authorities.

## Supplementary Information

**Additional file 1: Figure S1.** An overview of the CORDIALLY study. **Figure S2.** Patient disposition. **Figure S3.** (**A**) T2D study medication prescriptions by country, (**B**) patient enrolment by country and by HCP specialty (Prescribed Patient Set). **Table S1.** CORDIALLY study outcomes.

## Data Availability

The data underlying this article cannot be shared publicly due to privacy reasons of the participants. Researchers can request the data by submitting a reasonable proposal to the corresponding author.

## Abbreviations

AE: Adverse event
BMI: Body mass index
CABG: Coronary artery bypass graft
CEE: Central and Eastern Europe/European
CHF: Congestive heart failure
CKD: Chronic kidney disease
CV: Cardiovascular
CVD: Cardiovascular disease
CVOT: Cardiovascular outcomes trial
DPP4i: Dipeptidyl peptidase-4 inhibitor
eCRF: Electronic case report form
eGFR: Estimated glomerular filtration rate
GLP-1 RA: Glucagon-like peptide-1 receptor agonist
GP: General practitioner
HbA1c: Glycated haemoglobin
HCP: Healthcare professional
LVEF: Left ventricular ejection fraction
PAD: Peripheral arterial disease
PCI: Percutaneous coronary intervention
SD: Standard deviation
SE: Standard error
SGLT2i: Sodium-glucose cotransporter-2 inhibitor
T2D: Type 2 diabetes
UACR: Urine albumin creatinine ratio
