# Supplementary material for: Real-world characteristics, modern antidiabetic treatment patterns, and comorbidities of patients with type 2 diabetes in central and Eastern Europe: retrospective cross-sectional and longitudinal evaluations in the CORDIALLY® study
Source: Cardiovasc Diabetol. 2022 Oct 8;21:203. doi: 10.1186/s12933-022-01631-4 (PMC9548172; doi:10.1186/s12933-022-01631-4)
Supplement: Supplementary file 1 — Additional file 1: Figure S1. An overview of the CORDIALLY study. Figure S2. Patient disposition. Figure S3. (A) T2D study medication prescriptions by country, (B) patient enrolment by country and by HCP specialty (Prescribed Patient Set). Table S1. CORDIALLY study outcomes. [file 12933_2022_1631_MOESM1_ESM.docx]

**Supplementary Fig 1** An overview of the CORDIALLY study


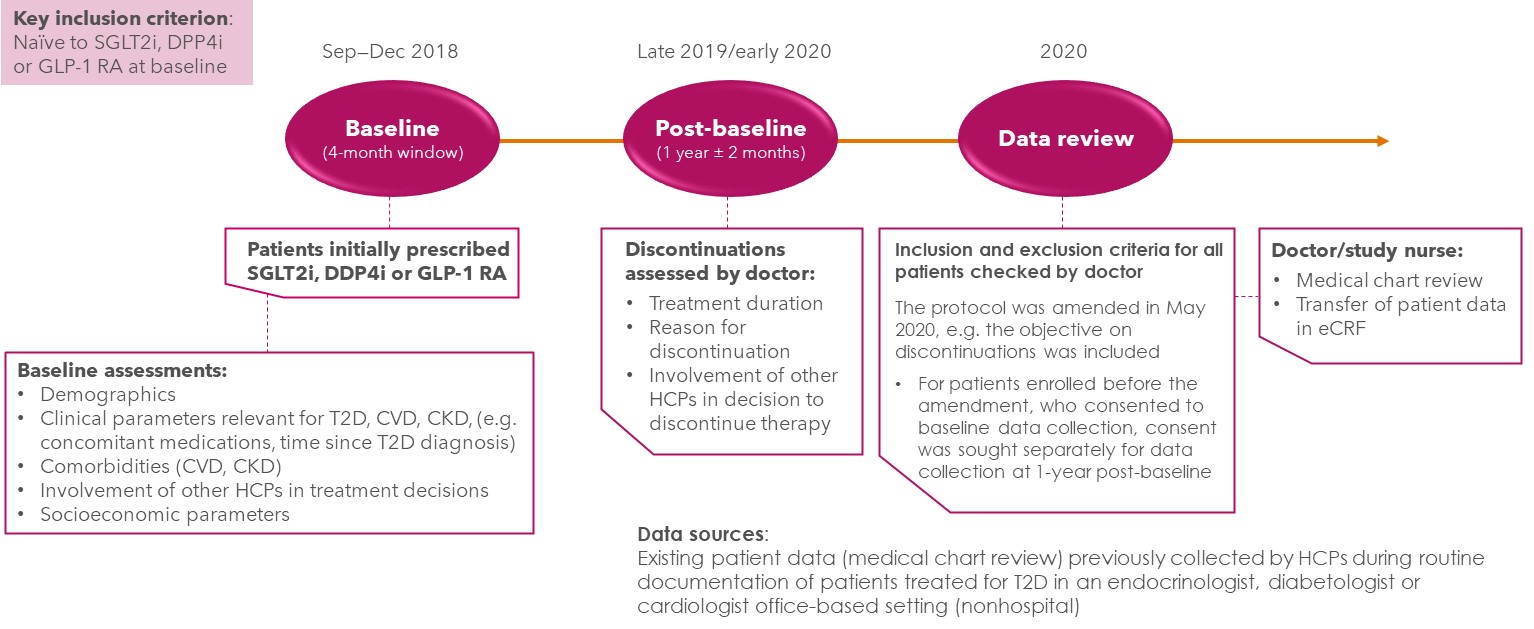
CKD, chronic kidney disease; CVD, cardiovascular disease; eCRF, electronic case report form; DPP4i, dipeptidyl peptidase-4 inhibitor; GLP-1 RA, glucagon-like peptide-1 receptor agonist; HCP, healthcare professional; SGLT2i, sodium-glucose cotransporter-2 inhibitor; T2D, type 2 diabetes.

**Supplementary Table 1** CORDIALLY study outcomes

| Outcome |  |
| --- | --- |
| Primary | *T2D patients’ baseline characteristics when initiating either empagliflozin or other SGLT2i, DPP4i, or GLP-1 RA on top of current antidiabetic treatment by different HCP specialties in Central Eastern European countries.*   1. Demographics (age, gender, height, weight, BMI, ethnicity*). 2. Time since T2D diagnosis 3. Clinical parameters relevant for T2D (HbA1c) 4. Clinical parameters relevant for CVD (blood pressure, LVEF, blood lipids) 5. Clinical parameters relevant for CKD (serum creatinine, eGFR, UACR) |
| Secondary | *Burden of comorbidities (prevalence of CVD, CKD, and risk factors) at baseline.*   1. Prevalence of comorbidities (AMI, cardiology intervention [PCI, CABG], IHD, heart failure, heart failure confirmed by echocardiography, stroke, PAD). 2. Risk factors:  - Overweight or obese - Hypertension (systolic ≥140 mmHg and/or diastolic ≥90 mmHg) - 10-year risk for fatal CVD according to the SCORE Risk Chart (considering age, gender, systolic blood pressure, smoking status, and total cholesterol) - Tobacco smoking (current/ex-smoker/never) - Physically inactive (less than 2.5 hours of moderate-intensity aerobic exercise or less than 75 minutes of vigorous aerobic exercise per week) - Family history of early onset heart disease - Family history of early onset kidney disease |
| Secondary | *Treatment use at baseline in patients with and without established CVD (defined as AMI, cardiology intervention, IHD, CHF, PAD, or stroke).*   1. T2D medication newly prescribed by the treating physician:  - SGLT2i (canagliflozin, dapagliflozin, empagliflozin, ertugliflozin) - DPP4i (alogliptin, linagliptin, sitagliptin, saxagliptin, vildagliptin) - GLP-1 RA (dulaglutide, exenatide, liraglutide, semaglutide, lixisenatide)  1. Reasons for choosing the treatment (rated as not relevant at all/moderately relevant/highly relevant):  - HbA1c lowering - Weight loss - Cardiovascular risk reduction - Favourable side effect profile - Simple dosing / administration - Guideline recommendation  1. Concomitant T2D medications:  - Metformin - Sulfonylurea - Acarbose - Pioglitazone - Insulin - Others  1. Concomitant CVD and CKD medications (class level only)  - Antihypertensives ACEi or ARBs - Statins - Low dose aspirin - Beta blockers - Diuretics |
| Secondary | *Association of socioeconomic parameters with treatment decisions at baseline.*   1. Employment status 2. Family status 3. Type of health insurance |
| Secondary | *Discontinuation rate, reasons for discontinuation, and duration of treatment with GLP-1 RA, DPP4i, and SGLT2i 1-year ± 2-months after baseline.*  Status of T2D therapy   1. Stop date, if available 2. Reasons for discontinuation:  - Investigator’s decision - Patient’s request (difficulties in medication handling) - Patient’s request (financial burden regarding co-payment) - Patient’s request (other) - Lost to follow-up - Adverse event - Death - Unknown - Other |

*Assessment of ethnicity (black or non-black) was necessary for calculation of eGFR.

ACEi, angiotensin converting enzyme inhibitors; AMI, acute myocardial infarction; ARB, angiotensin receptor blockers; BMI, body mass index; CABG, coronary artery bypass graft; CHF, congestive heart failure; CKD, chronic kidney disease; CVD, cardiovascular disease; DPP4i, dipeptidyl peptidase-4 inhibitor; eGFR, estimated glomerular filtration rate; GLP-1 RA, glucagon-like peptide-1 receptor agonist; HbA1c, glycated haemoglobin; HCP, healthcare professional; IHD, ischaemic heart disease; LVEF, left ventricular ejection fraction; PAD, peripheral arterial disease; PCI, percutaneous coronary intervention; SGLT2i, sodium-glucose cotransporter-2 inhibitor; T2D, type 2 diabetes; UACR, urine albumin-creatinine ratio.

**Supplementary Fig 2** Patient disposition


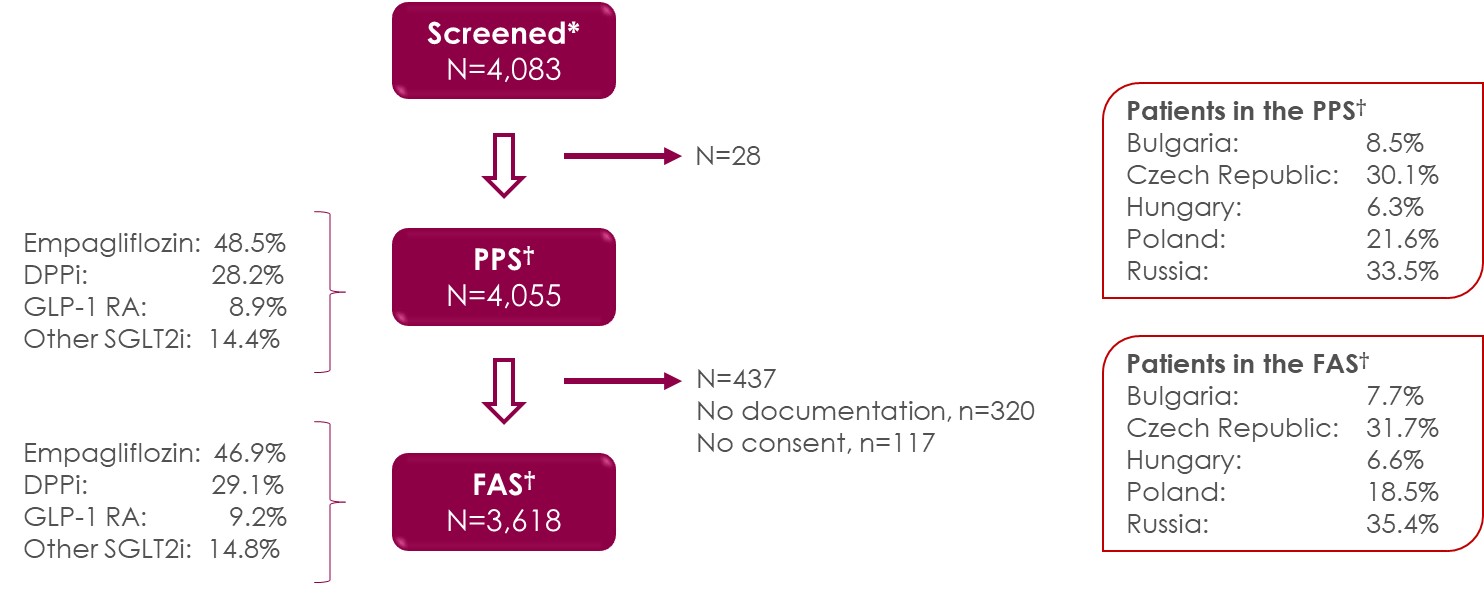


*Patients were screened at 177 sites in Bulgaria (15 sites), Czech Republic (58 sites), Hungary (10 sites), Poland (28 sites) and the Russian Federation (66 sites). †The PPS included patients with a first prescription of T2D study medication (baseline). The FAS included patients from the PPS with documentation at 1-year ± 2-months post-baseline. Discontinuations at 1-year ± 2-months post-baseline were analyzed with the FAS. All other analyses (all at baseline) used the PSS.

DPP4i, dipeptidyl peptidase-4 inhibitor; FAS, Full Analysis Set; GLP-1 RA, glucagon-like peptide-1 receptor agonist; HCP, healthcare professional; PPS, Prescribed Patient Set; SGLT2i, sodium-glucose cotransporter-2 inhibitor; T2D, type 2 diabetes.

.

**Supplementary Fig 3** (**A**) T2D study medication prescriptions by country, (**B**) patient enrolment by country and by HCP specialty (Prescribed Patient Set)


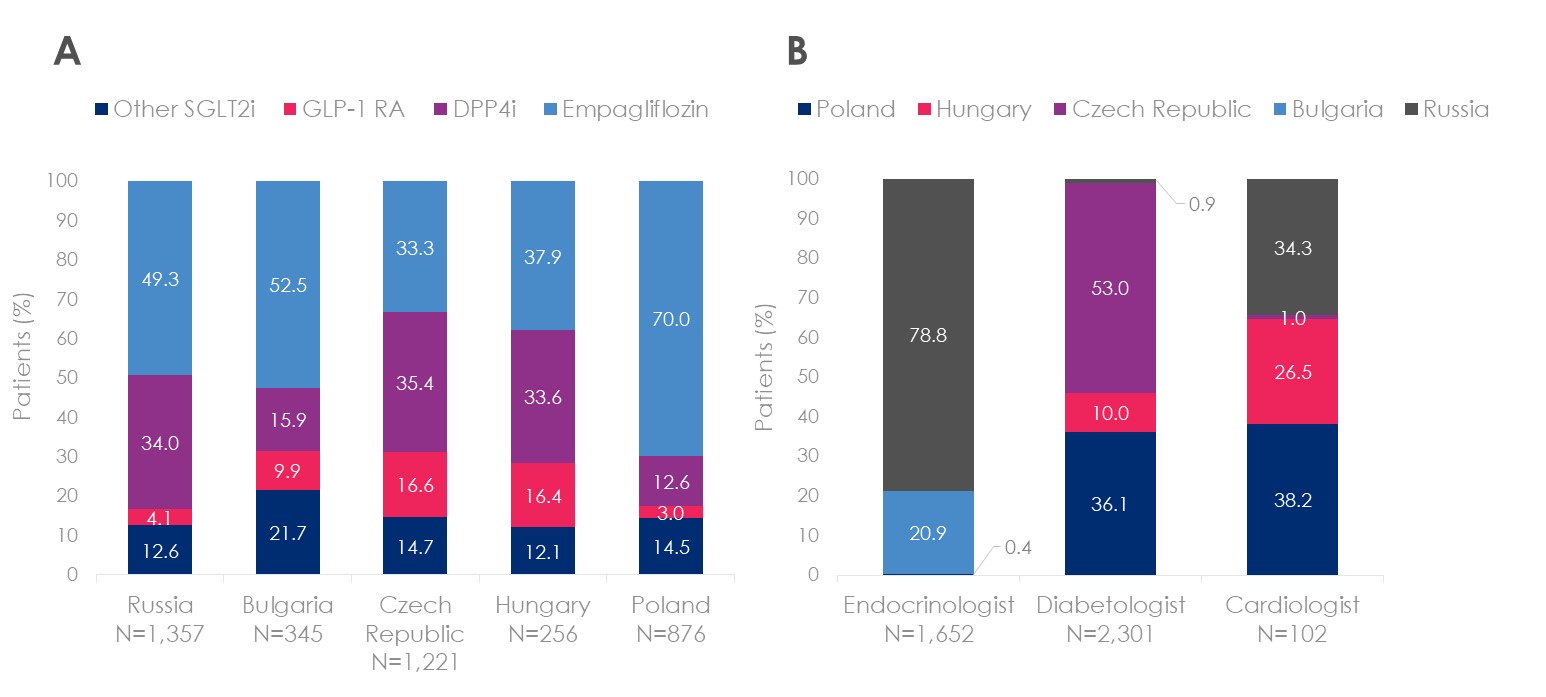
DPP4i, dipeptidyl peptidase-4 inhibitor; GLP-1 RA, glucagon-like peptide-1 receptor agonist; HCP, healthcare professional; SGLT2i, sodium-glucose cotransporter-2 inhibitor; T2D, type 2 diabetes.
